# Optimales neurologisches Outcome nach prolongierter Reanimation mit extrakorporaler Reanimation (eCPR)

**DOI:** 10.1007/s00101-024-01419-z

**Published:** 2024-06-07

**Authors:** Andreas Fichtner, Susanne Hiller, Sven Schönfelder, Peter Spieth

**Affiliations:** 1 Klinik für Anästhesie, Intensiv- und Notfallmedizin, Schmerztherapie und Palliativmedizin, Zeisigwaldkliniken Bethanien Chemnitz, Zeisigwaldstr. 101, 09130 Chemnitz, Deutschland; 2 Faculty of Medicine and University Hospital Carl Gustav Carus, TUD Dresden University of Technology, Dresden, Deutschland; 3 Zentrale Notfallaufnahme, Kreiskrankenhaus Freiberg, Freiberg, Deutschland; 4 DRF Luftrettung, Filderstadt, Deutschland; 5 Klinik für Anästhesiologie und Intensivtherapie, Universitätsklinikum Dresden an der TU Dresden, Dresden, Deutschland

## Anamnese

Wochentags um 7:45 Uhr stellte sich ein 41-jähriger Patient ohne relevante Vorerkrankungen aufgrund zunehmender Luftnot in der Notaufnahme eines Schwerpunktversorgers vor. Anamnestisch berichtete er über eine Pneumonie vor einer Woche; seit ein paar Tagen ginge er wieder arbeiten.

## Befund

Bei Aufnahme war der Patient kaltschweißig, zyanotisch mit einer Tachy‑/Orthopnoe und einer Atemfrequenz von 33/min. Auskultatorisch waren die Lungen seitengleich ohne pathologische Nebengeräusche belüftet. Die initiale S_p_O_2_ lag bei 79 % (Zusatzmaterial online). Es wurden umgehend eine NIV-Therapie mit PEEP und Druckunterstützung von jeweils 8 cm H_2_O und einer F_I_O_2_ von 0,5 initiiert sowie ein arterieller Zugang in die linke A. radialis gelegt. Die erste BGA zeigte eine Hypoxämie und eine relevante, v. a. metabolische Acidose. Das EKG ergab einen tachykarden Sinusrhythmus mit S1/Q3-Typ ohne Ischämiezeichen. Trotz verbesserter Oxygenierung unter der NIV-Beatmung kam es nach ca. 15 min zu einer Bradykardie mit Übergang in eine Asystolie, woraufhin umgehend mit einer leitliniengerechten kardiopulmonalen Reanimation (CPR) begonnen wurde.

## Diagnose

**Abb. 1:**
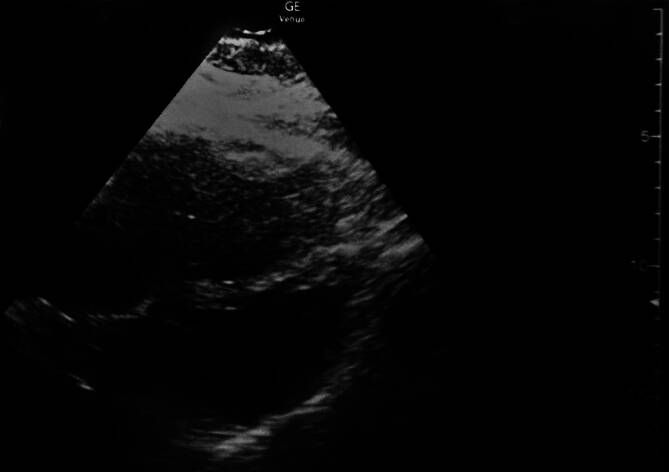
Notfallsonographiebefund mit „D sign“ des rechten Ventrikels

In der klinischen Untersuchung imponierte im Verlauf ein deutlich geschwollener Unterschenkel rechts. Echokardiographisch fand sich unter der Reanimation eine deutliche Rechtsherzbelastung mit Nachweis eines „D sign“ (Abb. 1), am rechten Bein ließ sich eine Thrombose der V. poplitea nachweisen. Klinisch wurde somit in Zusammenschau der Befunde eine fulminante Lungenembolie (LAE) mit akutem rechtsherzbedingtem kardiogenem Schock diagnostiziert.

## Therapie und Verlauf

Aufgrund nichtverfügbarer Alteplase wurde eine Notfalllysetherapie mit 10.000 IE Tenecteplase durchgeführt. Aufgrund der ausgeprägten Acidose erhielt der Patient mehrfach TRIS-Puffer. Nach ca. 30minütiger Reanimation konnte zunächst ein ROSC erreicht werden, jedoch blieb der Patient hypoton mit systolischen Blutdruckwerten um 60 mm Hg. Trotz hochdosierter kombinierter Katecholamintherapie mit Adrenalin, Dobutamin und Noradrenalin über einen inzwischen rechtsjugulär gelegten ZVK zeigte sich lediglich eine flache filiforme arterielle Pulskurve mit wiederholtem Abfall in einen funktionellen Kreislaufstillstand, sodass bis auf kurze Pausen im Minimal-ROSC eine nahezu kontinuierliche, frühzeitig maschinell unterstützte CPR (LUCAS®3, Physiocontrol, Redmond, WA, USA) durchgeführt wurde.

Auch 2 h nach der Lysetherapie mit inzwischen kontinuierlicher TRIS-Pufferung und maximaler Katecholamintherapie konnte bei ausgeprägtem Rechtherzversagen kein stabiler Kreislauf etabliert werden.

Bei prolongierter CPR wurden Prognose und die Gefahr der hypoxischen Hirnschädigung im Team diskutiert. Bei beobachtetem Herz-Kreislauf-Stillstand, unmittelbarem Reanimationsbeginn, gutem körperlichen Zustand des jungen Patienten sowie stets ausreichender Oxygenierung (S_p_O_2_ meist um 90 %) erschien, nach Rücksprache mit den-ECMO Team des Universitätsklinikums Dresden, die extrakorporale CPR (eCPR) mit dem Ziel des Bridging zur CT-Pulmonalisangiographie und ggf. Aspirationsthrombektomie als Therapie der potenziell reversiblen Ursache des Herz-Kreislauf-Stillstands als Ultima Ratio.

Nach telefonischer Absprache wurden bis zum Eintreffen des ECMO-Teams jeweils eine 6‑F-Schleuse ultraschallgestützt in die linksseitigen A. und V. femoralis eingelegt sowie eine invasive arterielle Druckmessung in der A. radialis dextra etabliert.

Komplizierend kam es bis zum Eintreffen des ECMO-Teams nach Lyse zur Sickerblutungen am jugulären ZVK, sodass bei relevantem Hb-Abfall auf 4,5 mmol/l 2 Blutkonserven transfundiert werden mussten.

Das inzwischen eingegangene Labor zeigte ein stark erhöhtes D‑Dimer von 15 mg/l sowie ein Troponin von 161 ng/l. Eine CT-Pulmonalisangiographie war bei anhaltender Kreislaufinstabilität und externer maschineller Herzdruckmassage nicht möglich.

**Abb. 2:**
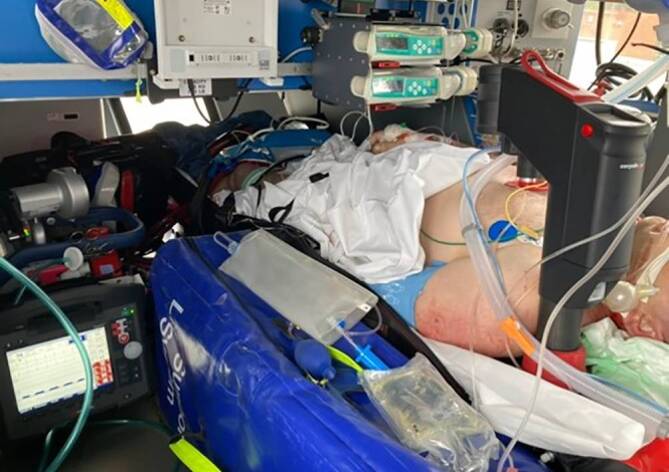
Verlasteter Patient mit ECLS und prophylaktisch installierter mechanischer Reanimationshilfe im für den ECMO-Transport umgebauten RTH vom Typ H135

Aufgrund der zeitkritischen Situation und der unmittelbaren Verfügbarkeit eines Primärhubschraubers (RTH) vom Typ H135 (Christoph 38) der DRF Luftrettung am Universitätsklinikum Dresden wurden Arzt und Pflegekraft des ECMO-Teams im Sinne eines schnellen Material- und Teamtransports eingeflogen. Sechs Minuten nach Eintreffen des ECMO-Teams und nach problemloser Kanülierung über die einliegenden Schleusen mittels 25 F/55 cm venöser Drainagekanüle und 17 F/23 cm arterieller Rückgabekanüle erfolgte um 11:00 Uhr die Etablierung der eCPR (Maquet Cardiohelp®, HLS 7.0, 3,5 l/min Blutfluss; 2 l/min Frischgasfluss; antegrade Beinperfusion über 6‑F-Schleuse A. femoralis sinistra). Unter diskreter Reduktion der Katecholamine und Beibehalten der kontinuierlichen Pufferung bei weiterhin bestehender schwerer Acidose konnte zügig die Transportfähigkeit hergestellt werden. Die zwischenzeitliche Anforderung mehrerer – wegen ausreichender Platzverhältnisse und spezieller Gerätefixierungsausrüstung für ECMO-Transporte vorgesehener – Sekundärhubschrauber (Intensivtransporthubschrauber, ITH) vom Typ H145 blieb erfolglos, ein bodengebundener Transport mittels ITW erschien aufgrund des zeitkritischen Geschehens nicht sinnvoll. Nach Rücksprache mit dem Piloten und HEMS-TC des noch auf dem Dachlandeplatz befindlichen RTH wurde dieser als in der beschriebenen Situation sinnvollste Transportoption für einen ECMO-Transport umgerüstet (Abb. 2). Da der Arzt des ECMO-Teams regulär auch als Notarzt der DRF Luftrettung auf dieser Station eingesetzt wird, waren die Anforderungen bezüglich luftfahrt- und medizintechnischer Einweisungen erfüllt. Unter persistierender hochdosierter Katecholamintherapie, mittlerweile noch stattgehabter Transfusion von insgesamt 4 Erythrozytenkonzentraten und kontinuierlichem ECLS-Support wurde der Patient zu weiterer Diagnostik und Therapie in das ECMO-Zentrum des Universitätsklinikums verlegt. Eine nach Aufnahme im Universitätsklinikum durchgeführte Pulmonalisangiographie zeigte nur noch Thrombusanteile auf Segment- und Subsegmentebene, sodass von einer Aspirationsthrombektomie Abstand genommen wurde. Unter Eindosierung von inhalativen NO und im weiteren Verlauf Sildenafil verbesserte sich die rechtsventrikuläre Funktion im Verlauf der nächsten 12 h, sodass die ECMO bereits am folgenden Tag dekanüliert und der Patient am 2. Tag nach dem Ereignis extubiert werden konnte. Nach passagerem Delir zeigten sich im weiteren Verlauf keine neurologischen Auffälligkeiten. Nach 9 Tagen auf der Intensivstation und weiteren 9 Tagen auf der Normalstation konnte der Patient bei Wohlbefinden und ohne neurologisches Defizit nach Hause entlassen werden.

## Diskussion

Dieser Fall zeigt aus unserer Sicht eindrücklich, dass auch nach prolongierter Reanimation – im Vergleich zu den aktuellen Empfehlungen verdreifachter Zeit [1] – bei beobachtetem Herz-Kreislauf-Stillstand suffizienten Reanimationsmaßnahmen und einer primär reversiblen Ursache des Ereignisses eine eCPR als Ultima Ratio erfolgreich eingesetzt werden kann [2]. Für die Durchführung der eCPR sprach in diesem Fall v. a. die unter Reanimation stets ausreichende Oxygenierung, die das Risiko einer hypoxischen Hirnschädigung vertretbar niedrig erscheinen ließ. Die unter kontinuierlichem lückenlosen Monitoring im Schockraum vom Zeitpunkt des funktionellen Kreislaufstillstands bis zum Beginn der eCPR minimierte No-Flow-Zeit hat sicher maßgeblich zum guten Outcome beigetragen. Zusätzliche, eine suffiziente CPR potenziell kompromittierende, Umlagerungs- und Transportmanöver wie bei einer präklinischen vergleichbaren Situation fanden nicht statt. Das interprofessionelle und klinikübergreifende Management der Reanimationsmaßnahmen in der Notaufnahme, die nach Indikationsstellung rasche Implantation der eCPR und der nachfolgende zügige Transport in das ECMO-Zentrum mit einer suffizienten Postreanimationsbehandlung waren aus unserer Sicht weitere Erfolgsfaktoren. Einzelne publizierte Fallberichte zeigen bei optimalen klinischen Bedingungen ebenfalls die vollständige Genesung nach 2 h Reanimation und ECLS bei Kammerflimmern [3], bzw. bei einem Kleinkind nach 1 h CPR bei vorbestehendem Herzfehler [4]. Bei präklinischem Kreislaufstillstand konnte ein akzeptables neurologisches Outcome hingegen in einem deutlich kürzeren Zeitraum von 30–40 min CPR bis ECLS gezeigt werden [5]. Die Kreislaufsituation war innerhalb der ersten 12 h nach dem Ereignis als sehr kritisch zu bewerten, erholte sich dann aber zügig. Die rechtsventrikuläre Funktion war in der letzten Kontrollechokardiographie vor der Entlassung wieder normwertig.

## Fazit für die Praxis

- Die Minimierung von Schnittstellen durch CRM-orientierte interprofessionelle und interdisziplinäre Teamarbeit kann es ermöglichen, bei beobachtetem Kreislaufstillstand auch mit deutlich prolongierter Reanimation und Zeit bis zur ECLS eine Restitutio ad integrum zu erreichen. Diesbezüglich relevante Punkte im vorgestellten Fall waren: unkomplizierte Übernahme des Primärhubschraubers (RTH) sowie dessen provisorische Umrüstung für den Rückweg, die überlappende Zusammenarbeit zwischen Notaufnahme-Team und ECMO-Team sowie die konsequente Minimierung der No-Flow-Zeit und erhaltene Oxygenierung.
- Für den Patiententransport mit ECLS kann im Notfall auch ein sonst nicht dafür geeigneter Primärhubschrauber ausreichend sein.

## Supplementary Information

Verlauf der Vital- und POCT-Werte während der Primärversorgung
