# Erratum zu: Optimales neurologisches Outcome nach prolongierter Reanimation mit extrakorporaler Reanimation (eCPR)

**DOI:** 10.1007/s00101-025-01560-3

**Published:** 2025-07-14

**Authors:** Andreas Fichtner, Susanne Hiller, Sven Schönfelder, Peter Spieth

**Affiliations:** 1 Klinik für Anästhesie, Intensiv- und Notfallmedizin, Schmerztherapie und Palliativmedizin, Zeisigwaldkliniken Bethanien Chemnitz, Zeisigwaldstr. 101, 09130 Chemnitz, Deutschland; 2 Faculty of Medicine and University Hospital Carl Gustav Carus, TUD Dresden University of Technology, Dresden, Deutschland; 3 Zentrale Notfallaufnahme, Kreiskrankenhaus Freiberg, Freiberg, Deutschland; 4 DRF Luftrettung, Filderstadt, Deutschland; 5 Klinik für Anästhesiologie und Intensivtherapie, Universitätsklinikum Dresden an der TU Dresden, Dresden, Deutschland

**Erratum zu:**

**Anaesthesiologie 2024**

10.1007/s00101-024-01419-z

Die Zweitaffiliation von Prof. Dr. med. habil. Andreas Fichtner, MME – „Faculty of Medicine and University Hospital Carl Gustav Carus, TUD Dresden University of Technology, Dresden, Germany“ – wurde in diesem Artikel ergänzt. Der Originalbeitrag wurde entsprechend korrigiert.

