# Supplementary material for: Optimal neurological outcome after prolonged resuscitation with extracorporal life support (eCPR)
Source: Anaesthesiologie. 2024 Jun 7;73(6):417–20. [Article in German] doi: 10.1007/s00101-024-01419-z (PMC11164717; doi:10.1007/s00101-024-01419-z)

Zusatzmaterial zum Beitrag **„Optimales neurologisches Outcome nach prolongierter Reanimation mit eCPR“** von Fichtner A, Hiller S, Schönfelder S und Spieth P (2024) in *Die Anaesthesiologie*
Beitrag und Zusatzmaterial stehen Ihnen auf www.springermedizin.de zur Verfügung. Bitte geben Sie dort den Beitragstitel in die Suche ein.

Verlauf der Vital- und POCT-Werte während der Primärversorgung.

Verkomplizierend war das BGA-Gerät in der ZNA zwischenzeitlich nicht einsatzfähig, sodass die Proben teilweise auf der Intensivstation analysiert werden mussten. Vereinzelte nicht generierte Werte wurden als Trendlinie dargestellt.

<u>Vitaldaten aus dem PDMS</u>

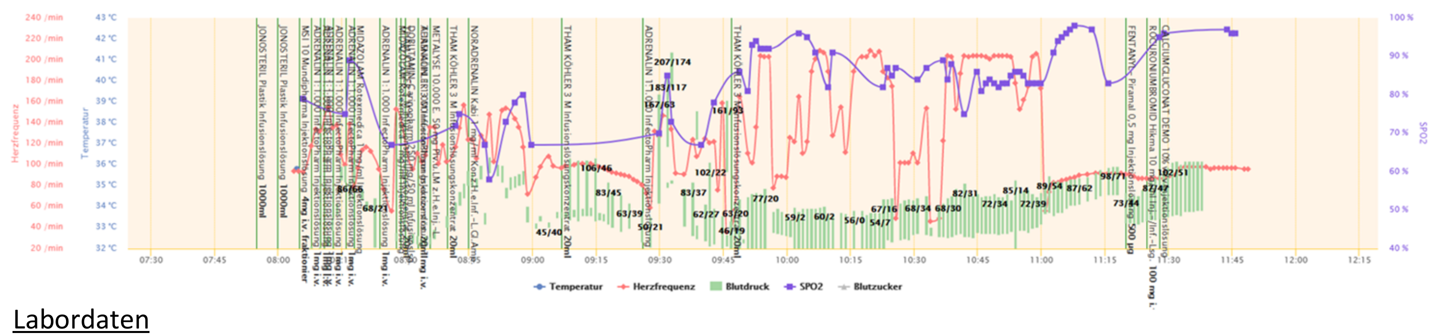

<u>Labordaten</u>

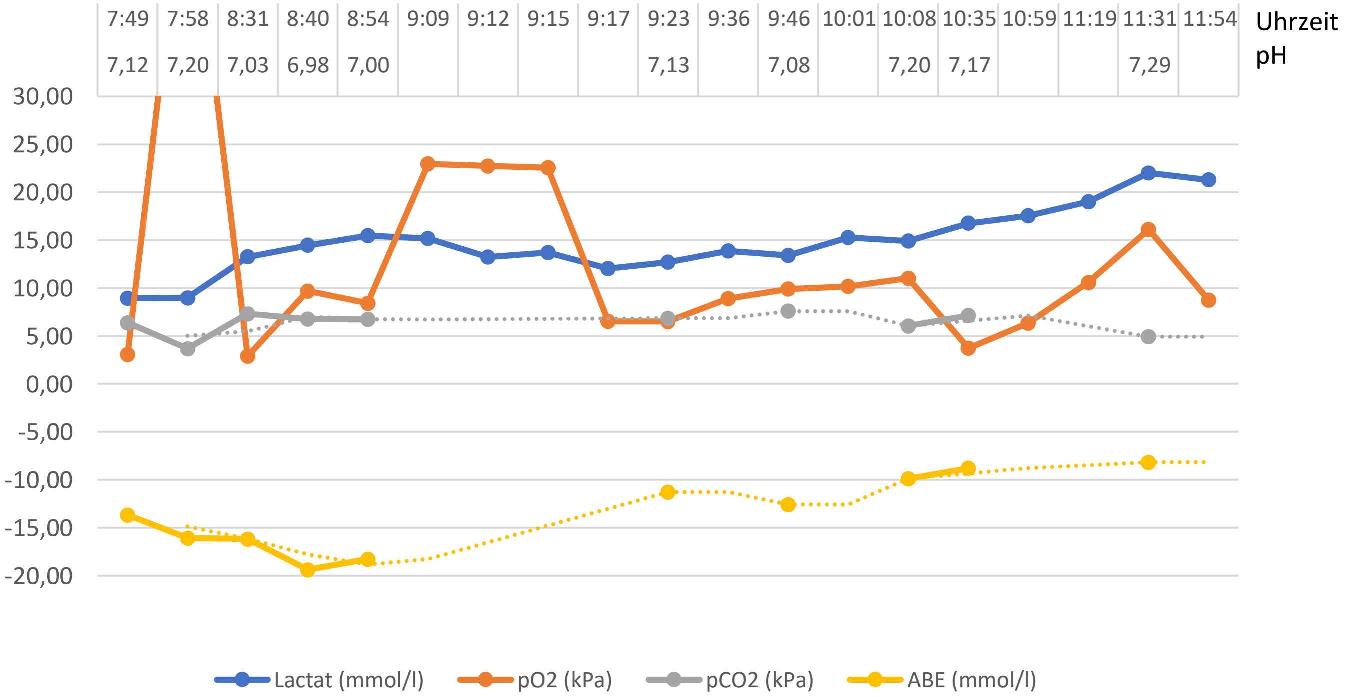

Supplement: Supplementary file 1 — Verlauf der Vital- und POCT-Werte während der Primärversorgung [file 101_2024_1419_MOESM1_ESM.pdf]
